# Functional and genetic analyses of *ZYG11B* provide evidences for its involvement in OAVS

**DOI:** 10.1002/mgg3.1375

**Published:** 2020-08-01

**Authors:** Angèle Tingaud‐Sequeira, Aurélien Trimouille, Sandrine Marlin, Estelle Lopez, Marie Berenguer, Souad Gherbi, Benoit Arveiler, Didier Lacombe, Caroline Rooryck

**Affiliations:** ^1^ Maladies Rares: Génétique et Métabolisme (MRGM) U 1211 INSERM Univ. Bordeaux Bordeaux France; ^2^ Service de Génétique Médicale Centre de Référence Anomalies du Développement et Syndromes Malformatifs CHU de Bordeaux Bordeaux France; ^3^ Département de Génétique Centre de Référence des Surdités Génétiques Institut Imagine Hôpital Universitaire Necker‐Enfants‐Malades Paris France; ^4^ Institut Imagine U 1163 INSERM Université Paris Descartes Paris France

**Keywords:** craniofacial anomalies, etiology, genetics, Goldenhar, hemifacial microsomia, OAVS, ubiquitine ligase, wavy notochord, ZYG11B

## Abstract

**Background:**

The Oculo‐Auriculo‐Vertebral Spectrum (OAVS) or Goldenhar Syndrome is an embryonic developmental disorder characterized by hemifacial microsomia associated with auricular, ocular and vertebral malformations. The clinical heterogeneity of this spectrum and its incomplete penetrance limited the molecular diagnosis. In this study, we describe a novel causative gene, *ZYG11B*.

**Methods:**

A sporadic case of OAVS was analyzed by whole exome sequencing in trio strategy. The identified candidate gene, *ZYG11B*, was screened in 143 patients by next generation sequencing. Overexpression and immunofluorescence of wild‐type and mutated ZYG11B forms were performed in Hela cells. Moreover, morpholinos were used for transient knockdown of its homologue in zebrafish embryo.

**Results:**

A nonsense de novo heterozygous variant in *ZYG11B*, (NM_024646, c.1609G>T, p.Glu537*) was identified in a single OAVS patient. This variant leads in vitro to a truncated protein whose subcellular localization is altered. Transient knockdown of the zebrafish homologue gene confirmed its role in craniofacial cartilages architecture and in notochord development. Moreover, *ZYG11B* expression regulates a cartilage master regulator, *SOX6*, and is regulated by Retinoic Acid, a known developmental toxic molecule leading to clinical features of OAVS.

**Conclusion:**

Based on genetic, cellular and animal model data, we proposed *ZYG11B* as a novel rare causative gene for OAVS.

## Graphical abstract

The Oculo‐Auriculo‐Vertebral Spectrum or Goldenhar Syndrome is an embryonic developmental disorder characterized by hemifacial microsomia associated with auricular, ocular and vertebral malformations. Its etiology is poorly characterized. In this study, we describe a novel causative gene, ZYG11B.

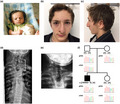

## 1 INTRODUCTION

The Oculo‐Auriculo‐Vertebral Spectrum (OAVS) or Goldenhar Syndrome (GS) or Hemifacial Microsomia [MIM: 164210] is a rare developmental disease whose incidence is around 1/26 500 (Barisic et al., 2014). It is the second most frequent embryonic malformative spectrum of head and neck. The OAVS includes various malformations involving structures derived from the first and second branchial arches, mainly ears, eyes, mandible and vertebrae. The clinical phenotype is highly heterogeneous and characterized by hemifacial microsomia and/or asymmetric ear anomalies (microtia, preauricular tags, external auditory canal atresia, conductive and/or sensorineural hearing loss…) and/or ocular defects (epibulbar dermoids, upper eyelid coloboma, microphtalmia…), and/or vertebral malformations (fused cervical vertebrae, vertebral puzzle…). Other features are cleft lip and/or palate, cardiac, renal or cerebral malformations, and rarely mental deficiency (Gorlin, Cohen, & Hennekam, 2001). OAVS was first described in 1952 by Maurice Goldenhar (Goldenhar, 1952) and the clinical spectrum of this disorder was then expanded (Gorlin et al., 2001; Gorlin, Jue, Jacobsen, & Goldschmidt, 1963).

Nongenetic causes are hypothesized for this spectrum, including maternal diabetes (Wang, Martínez‐Frías, & Graham, 2002), placental vascular disruption (Poswillo, 1975) and exposure to toxic substances during pregnancy such as retinoic acid (RA). Indeed, RA human embryopathies present features overlapping with OAVS phenotypes (Coberly, Lammer, & A. M., 1996; Glineur et al., 1999; Lammer et al., 1985; Mondal, Shenoy, & Mishra, 2017). The genetic origin of OAVS is supported by the description of familial cases (Stoll, Viville, Treisser, & Gasser, 1998; Vendramini‐Pittoli & Kokitsu‐Nakata, 2009), and the identification of diverse chromosomal aneuploidies and few recurrent CNVs (Copy Number Variants) such as deletion 22q11.2 (Derbent et al., 2003; Dos Santos et al., 2014; Xu, Fan, & Siu, 2008) and duplication 4p16.1 (Barber, 2018; Beleza‐Meireles et al., 2015; Bragagnolo et al., 2018) in OAVS patients. Moreover, the identification of *MYT1* as the first causative gene in OAVS confirms the genetic etiology (Berenguer et al., 2017; Lopez et al., 2016). Of note, recently in *AMIGO2*, one *de novo* nonsense variant was found associated with OAVS. These previously described cases are in favor of a dominant autosomal transmission with incomplete penetrance.

Identification of new genes associated with OAVS is a crucial step in understanding the physiopathology of the disease. In this aim, we performed exome sequencing in other OAVS patients and identified a novel causative gene named *ZYG11B* (zyg‐11 family member B, cell cycle regulator).

## 2 METHODS

### 2.1 Patient

**Figure 1:**
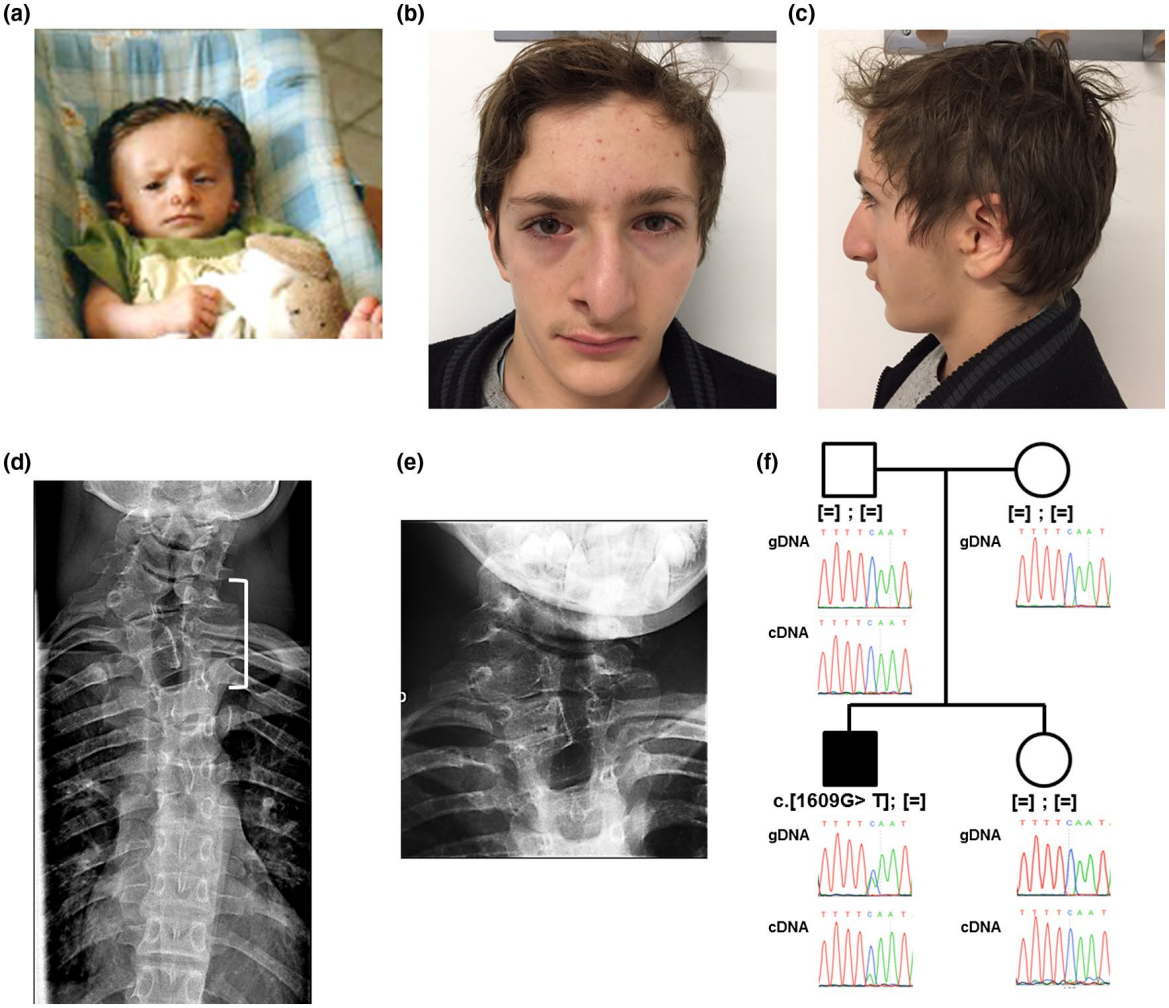
Photographs and pedigree of the patient carrying the c.1609G>T, p.(Gly537*) variant in *ZYG11B*. (a–c) Photographs showed the proband at infant stage (a) and at 15 years (b and c). (d) X‐ray imaging of patient vertebrae showing vertebral fusion of hypoplastic C7‐D1 vertebrae (white bracket). (e) Enlarged of C7‐D1 vertebrae. (f) Family tree and electrophoregrams showing the de novo heterozygous nonsense variant in the proband. Sequencing of RT‐PCR amplicons revealed that the transcript escapes to nonsense‐mediated RNA decay

The proband is a male patient. He is the first child of an unrelated couple without medical history. He was born at term after an uneventful pregnancy. No psychomotor delay was observed along his development. He presented with right hemifacial microsomia involving mandibular hypoplasia. Auricular anomalies included left microtia (grade 3), bilateral preauricular tags and bilateral dysplasia with polyotia. Audiometric tests were normal. Ocular anomalies were left epibulbar dermoid and right eyelid coloboma (Figure 1a–c). Vertebral fusion of hypoplastic C7‐D1 vertebrae was observed (Figure 1d,e). In addition, costal agenesis, right nostril hypoplasia with pit and tag, bilateral cheek pits, high arched palate and 2–3 toe syndactylies were noted. Renal ultrasonography and echocardiography were normal. Neither transfrontal echography nor magnetic resonance imaging of the brain showed cerebral anomalies.

Exome sequencing. Written informed consent for genetic studies was obtained prior to collecting blood samples for DNA extraction. Moreover, consent was obtained from the patient's parent for the publication of identifying images in an online open‐access publication. The local ethics committee (Comité de Protection des Personnes: DC2012/76) approved this study. Library preparation, exome capture, sequencing and data analysis were performed by IntegraGen SA, using SureSelect Human All Exon kit V2 in‐solution enrichment methodology (Agilent), followed by paired‐end 75 bases massively parallel sequencing on Illumina HiSeq2000 (Illumina). Variants were filtered using Eris software (IntegraGen), with a minimal read depth of 10x. Only rare variants (allele frequency <0.1% in public databases GnomAD, Exac and EVS), with a potential effect on proteins (nonsynonym, and synonym or intronic variants within 5 bp of a splice site), and segregating with a possible Mendelian inheritance in the trio were kept for analysis.

Cohort screening for *ZYG11B* mutations (ZYG11B Ref Seq: NM_024646/NP_078922). We performed screening for other nucleotide variants of *ZYG11B* in 143 OAVS patients. Exons, exon–intron boundaries, 5′UTR and 3′‐UTR (transcript ENST00000294353.6, http://www.ensembl.org/) were amplified using a standard protocol. Seventeen primer pairs were designed (under request). PCR fragments were sequenced by NGS (GS Junior, Roche or MiSeq, Illumina) and Sanger sequencing. The online tool SureDesign provided by Agilent was used for RNA probes design targeting *ZYG11B* exons, exon–intron junctions, 5′‐UTR and 3′‐UTR (ENSG00000162378, http://www.ensembl.org). After library preparation, the SureSelect QXT kit (Agilent) was used for the capture step following the manufacturer's instructions. Sequencing was performed on MiSeq sequencer from Illumina using the MiSeq Reagent Kit v3 (Agilent) for paired‐end sequencing. Alignment and variant calling was processed with the MiSeq Reporter Software (Illumina).

### 2.2 Expression plasmid

All primers used for cloning experiments are listed in Table S1. The construct including *ZYG11B* wild‐type cDNA in pCS2+ vector was named ZYG11B‐WT and the one carrying the variant c.1609G>T was named ZYG11B‐E537*.

### 2.3 Cell culture

For All‐Trans RA (ATRA, Sigma Aldrich) experiments, cells were treated for 48 hr with 0.1 mM of ATRA. For siRNA experiments, cells were transfected with 20nM of control nonspecific siRNA (EHUEGFP, Sigma) or with specific siRNA targeting *ZYG11B* (EHU022091, Sigma) using DharmaFECT™ Transfection Reagent following manufacturer's instructions (Dharmacon Horizon Discovery). Cell proliferation was assessed by Crystal Violet staining.

### 2.4 Quantitative real‐time PCR

Total RNA extraction and reverse‐transcription were processed as previously described (Lopez et al., 2016). Primers used are listed in Table S1. The relative transcript level was calculated as fold change using the 2^−ΔΔCt^ method.

### 2.5 Immunodetection

Immunocytochemistry and Western blot were performed with primary antibody targeting ZYG11B (HPA028156, SIGMA, diluted 1/300).

### 2.6 Zebrafish zyg11 knockdown

Zebrafish were produced in our facilities, in accordance with the French Directive (Ministère de l'Agriculture) and in conformity with the European Communities Council Directive (2010/63/EU). Morpholino oligonucleotides (MOs) were designed as complementary to the sequence flanking the translation initiation codon of *zyg11* (MO‐ATG: 5′‐TTGAGAAAGATGGATCATAACGGCC‐3′). At 5 dpf, embryos were stained using Alcian blue/Alizarin red stain according to Walker and Kimmel (https://wiki.zfin.org).

## 3 RESULTS

WES was performed in trio strategy, identifying a heterozygous nonsense *de novo* variant (chr.1 g.52801942G>T, c.1609G>T, p.(Gly537*)) in *ZYG11B* not reported in any database. A total of 30 reads (16+, 14−) out of 59 identified the variant which was confirmed by Sanger sequencing (Figure 1f). The pLI factor of *ZYG11B* is 1 according to GnomAD database, with an observed/expected loss‐of‐function variants score of 0.11 (90% CI at 0.05–0.26), in favor of an essential biological function. After exome filtering, six additional variants were considered: due to their segregation, frequencies in GnomAD (>1%), genes’ pLI, and genes function, all these variants were discarded. Nonsense RNA decay was excluded as patient's leucocytes expressed mutated transcripts (Figure 1f). *ZYG11B* was screened in 143 OAVS patients, without identifying any other pathogenic variant (Supplementary methods).

**Figure 2:**
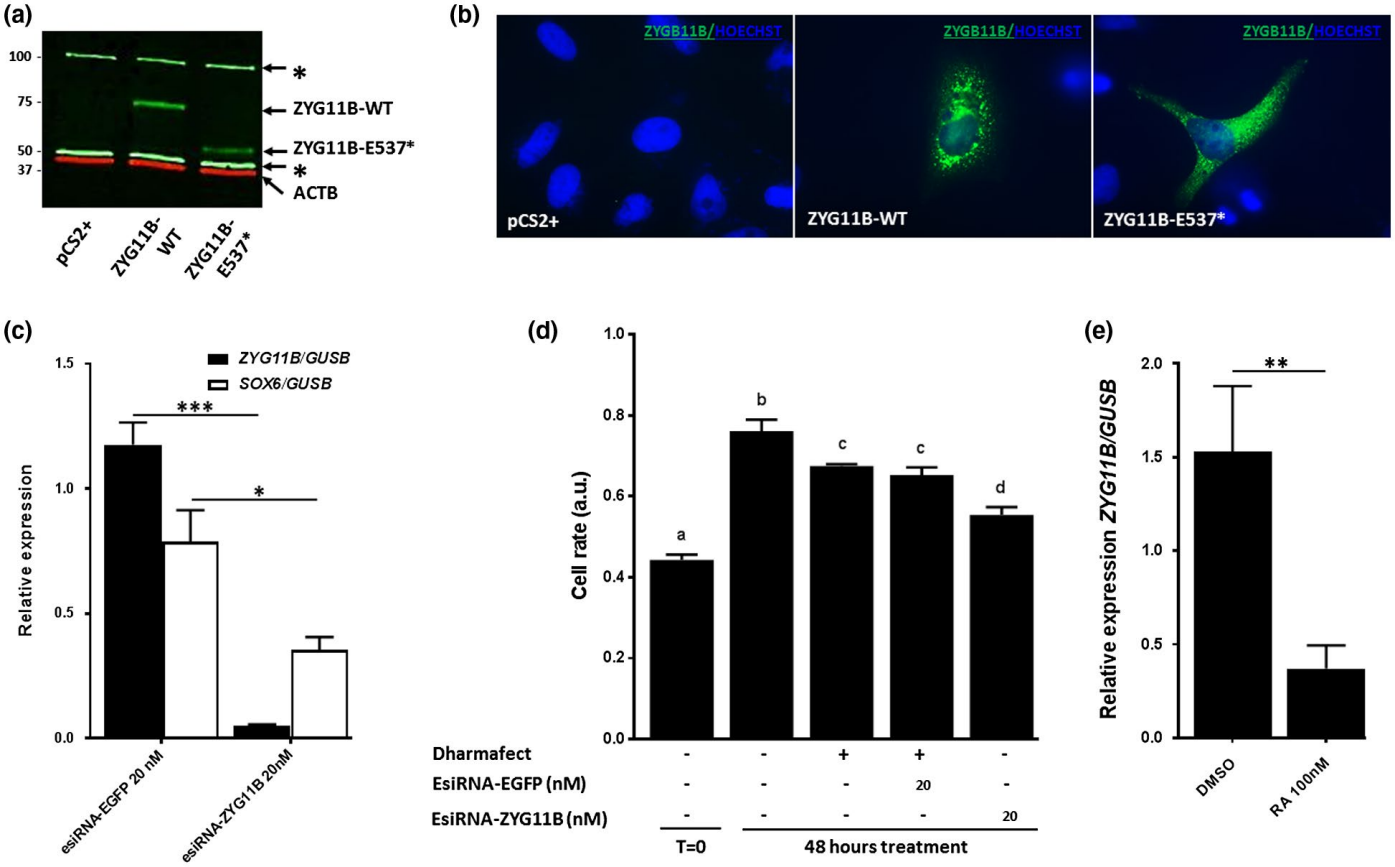
The nonsense variant encodes a mislocalized truncated ZYG11B protein. (a) Western blot validated overexpression experiments. (b) Immunocytochemistry detection of ZYG11B‐WT and ZYG11B‐E537* in HeLa cells. (c) RT‐qPCR experiments validated *ZYG11B* expression knockdown by the specific siRNA‐ZYG11B which also induced a significant down‐regulation of *SOX6* expression, *t* test *p* < .05. (d) Cell proliferation is significantly decreased when treated with siRNA‐ZYG11B (Kruskal–Wallis test followed by Dunns’ post hoc multiple comparison test (*p* < .05)). (d) RT‐qPCR analysis of *ZYG11B* expression showed a down‐regulation following RA treatment in HeLa cells (*t* test *p* < .05)

Although *ZYG11B* transcripts were detected in HeLa cells, Western blot analysis was not sensitive enough to detect endogenous ZYG11B protein. However, overexpression of ZYG11B‐WT or ZYG11B‐E537* was strongly detected in transfected cells. As expected, the nonsense variation produced a truncated protein of lower molecular weight, ≈50kDa instead of ≈75kDa (Figure 2a). Immunodetection of overexpressed ZYG11B‐WT revealed a vesicular, concentric and perinuclear signal whereas ZYG11B‐E537* presented also a vesicular signal but diffuse in all the cytoplasm (Figure 2b).

Based on haplo‐insufficiency hypothesis, inhibition of *ZYG11B* expression was tested in cell and animal models. We then performed siRNA experiments targeting specifically endogenous *ZYG11B* transcripts as demonstrated by RT‐qPCR experiments (Figure 2c). We investigated if the regulation of *ZYG11B* expression would alter *SOX6* expression, as it is a master regulator involved in cartilage development. Indeed, a strong down‐regulation of *SOX6* gene expression was observed (Figure 2c). Choosing cell proliferation as an endpoint to highlight potential cell cycle deregulation, cell rate was measured at t = 0, just before transfection, and after 48 hr of transfection. A significant lower proliferation rate was observed with the specific siRNA versus control siRNA (Figure 2d).

T‐qPCR experiments demonstrated that *ZYG11B* endogenous expression in HeLa cells can be down‐regulated by RA treatment for 48 hr (Figure 2e). However, *MYT1* overexpression for 24 hr did not affect *ZYG11B* endogenous expression (Figure S1).

**Figure 3:**
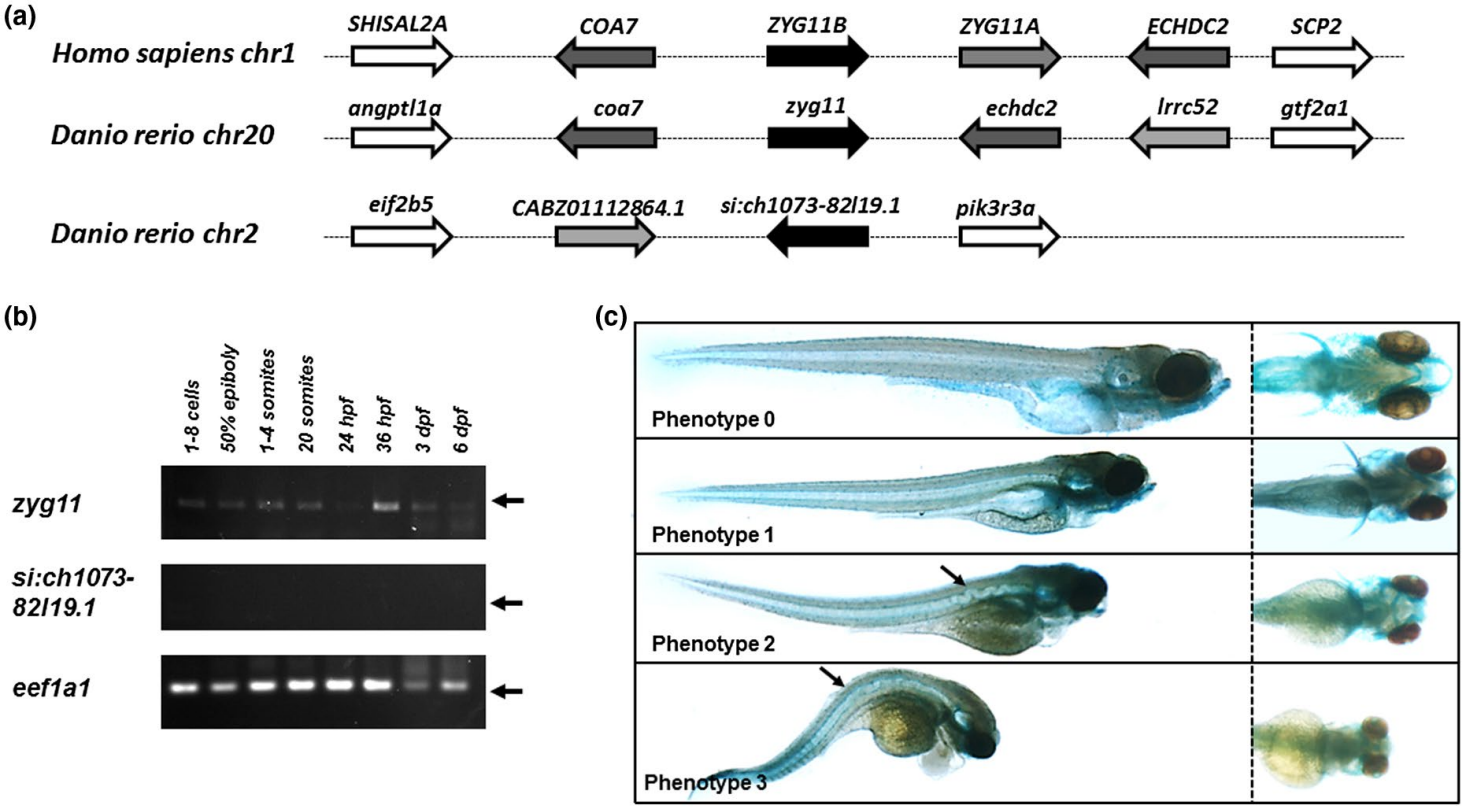
Zebrafish *zyg11* gene knockdown impaired craniofacial cartilages and notochord development. (a) Conserved synteny between human *ZYG11B* genomic environment and zebrafish homologues. (b) expression pattern of *zyg11* and *si:ch1073‐82l19.1* genes in zebrafish by RT‐PCR. (c) At 120 hpf, embryos were classified in 4 phenotypes

We then investigated the developmental role of *ZYG11B* in zebrafish model. Two *ZYG11B* homologues were identified, *zyg11* and *si:ch1073‐82l19.1* consistent with the known whole genome duplication in teleost (Figure 3a). Developmental expression study showed that *zyg11* was significantly expressed from early stages and throughout zebrafish embryogenesis whereas *si:ch1073‐82l19.1* was not (Figure 3b). Transient knockdown was then only performed for *zyg11* with MO‐ATG at 2 ng per embryo. In order to assess the involvement of *zyg11* in cartilage development, embryos were stained with Alcian Blue at 5dpf. Of note, preliminary experiments to discard nonspecific effects due to activation of p53‐dependent apoptosis were performed through coinjection of MO specifically targeting p53 transcripts (data not shown). At this stage, four phenotypes were defined (Figure 3c): (a) phenotype 0 was defined as wild‐type embryo; (b) phenotype 1 was defined as embryo presenting altered architecture of craniofacial cartilages and microphtalmia; (c) phenotype 2 was defined as phenotype 1 plus a wavy notochord in the anterior part; (d) phenotype 3 was defined as globally strongly altered phenotype with major curved tail probably corresponding to nonspecific phenotype. Contrary to control groups, in the group injected with MO 2 ng/embryo, 25% of embryos presented phenotype 1 and 25% phenotype 2. A very small number of embryos presented with phenotype 3. These results confirmed a specific role of *zyg11* in head development and specifically craniofacial cartilages and eye development. In phenotypes 2 and 3, craniofacial cartilage alterations include flattening of ceratohyal cartilage, abnormal distance between the ceratohyal and meckel's cartilages, abnormal angulation between the ceratohyal and palatoquadrate cartilages and abnormal angulation between the two ceratohyal cartilages in phenotype 3, craniofacial cartilages were almost absent (Figure 3c). Interestingly, those animals with phenotype 3 from injected group at MO 2 ng/embryo also presented partial wavy notochord as for phenotype 2 embryos (Figure 3c). Significant higher rates of phenotypes 2 and 3 were observed on MO‐AUG injected group.

We intended to perform rescue experiments in zebrafish model with human ZYG11B‐WT and ZYG11B‐E537* forms by coinjecting their cRNA and MO‐ATG targeting *zyg11*. Unfortunately, recovery of the MO‐induced phenotype by wild‐type form was not significantly observed. It could be explained by evolutionary differences. Indeed, in human, tandem duplication arose leading to *ZYG11A* and *ZYG11B* whereas in zebrafish genome *zyg11* and *si:ch1073‐82l19.1* arose from the whole genome duplication described in teleosts (Figure 3a). So, two independent events are noticed in Human and zebrafish genomes probably diluting the heterologous rescue capacity.

## 4 DISCUSSION

ZYG11B protein is characterized as a member of an E3 ubiquitin ligase complex and is involved in substrate recognition for subsequent proteasome degradation. It is a multi‐domain protein including a Von Hippel–Lindau‐box, three leucine‐rich repeat motifs and an armadillo helical domain (ARM). ZYG11B is demonstrated to be associated with Cullin‐2, an E3 ubiquitin ligase, in several models as *C. elegans* or animal and human cell lines (Balachandran et al., 2016; Liu, Vasudevan, & Kipreos, 2004; Yamaki, Kagawa, Hatta, Natsume, & Kawahara, 2016). In addition to the anaphase‐promoting complex/cyclosome (APC/C), CUL2/ZYG11 complex is necessary for meiosis completion at metaphase to anaphase transition at meiosis II (Liu et al., 2004; Sonneville & Gönczy, 2004). Moreover, ZYG11B through the CUL2‐based complex is also demonstrated to be involved in cell differentiation through the Patched1 receptor (Ptcd1), a twelve transmembrane domain receptor of Shh (Yamaki et al., 2016). Thus far, there is no evidence of *ZYG11B* involvement in developmental disorders. Identification of a de novo nonsense variant in *ZYG11B* in the exome of an OAVS patient was then interesting. Unfortunately, we do not find any additional variant in 143 OAVS patients. This finding is not surprising as genetic heterogeneity is highly suspected in OAVS: we only identified few variants in the first OAVS gene *MYT1* (Berenguer et al., 2017; Lopez et al., 2016).

The *ZYG11B* variant produces *in vitro* a truncated protein whose intracellular localization is altered, as demonstrated by overexpression experiments. Potential pathogenicity of the variant could then be linked to these data. Moreover, knockdown of *ZYG11B* expression induces down‐regulation of cellular proliferation, maybe linked to cell cycle control, a complementary role of *ZYG11A* and *ZYG11B* previously demonstrated (Balachandran et al., 2016).

RA is a toxic environmental agent causing OAVS features (Coberly et al., 1996; Lammer et al., 1985) and as the *MYT1*‐RA relationship was previously hypothesized (Berenguer et al., 2017; Lopez et al., 2016), investigation of the relationship with *ZYG11B* was performed. *ZYG11B* expression appears down‐regulated by cellular RA exposure.

To support the involvement of *ZYG11B* in craniofacial development, we then produced a zebrafish model of *zyg11* knockdown. Interestingly, embryos are strongly impacted at the craniofacial level, based on observation of the well‐described meckel‐palatoquadrate‐basihyal‐ceratohyal‐hyosymplectic architecture. Interestingly, a wavy notochord phenotype is also observed. Wavy notochord has already been observed in genetic or toxic zebrafish models of copper deficiency (Mendelsohn et al., 2006; Raldúa & Babin, 2007). Indeed, in zebrafish *atp7a* mutant *calamity* or *atp7a* knockdown models present with a wavy notochord rescuable by exogenous copper. Moreover, environmental copper chelating agents are identified based on observation of wavy notochord as BLT‐1 (Raldúa & Babin, 2007; Sandoval et al., 2013) or kalihinol F (Sandoval et al., 2013). This could be an additional clue for a synergic role of environmental and genetic factors leading to the same phenotype.

In summary, we describe a *de novo* nonsense variant in *ZYG11B*, associated with OAVS phenotype. *In vitro* studies show that the resulting truncated protein presents a subcellular mislocalization. Moreover, *ZYG11B* belongs to RA regulated transcriptome, such as the first OAVS gene *MYT1* (Lopez et al., 2016). Furthermore, in zebrafish model, *zyg11* is involved in craniofacial cartilage and notochord development. Taken together, and considering the molecular heterogeneity described in this spectrum, these findings support *ZYG11B* as a new causative gene for OAVS.

The only reported genes associated with OAVS are *MYT1* (Berenguer et al., 2017; Lopez et al., 2016), *AMIGO2* (Rengasamy Venugopalan et al., 2019) and *ZYG11B* (in the present study). All these genes are poorly characterized and their developmental functions are not fully understood. Moreover, their known functions are different. Molecular hypotheses for OAVS physiopathology are still difficult to highlight. More data and candidate genes are needed to isolate a common biological pathway involved in OAVS.

## CONFLICT OF INTERESTS

The authors declare no conflict of interest.

## AUTHOR CONTRIBUTIONS

AT‐S, AT and CR conceived and designed the experiments. AT‐S, EL, MB, SG, and MB performed the experiments. AT‐S, AT, SM, EL, and CR analyzed the data. AT‐S, AT, CR wrote the paper. SM, BA, DL, and CR edited, revised and approved the final version of the manuscript.

## Supporting information

Table S1‐Fig S1

## Data Availability

The identified variant (NM_024646, c.1609G>T) was submitted to ClinVar database (submission identification: SUB6959693). All data that support the findings of this study are available on request from the corresponding author.

## References

[mgg31375-bib-0001] Balachandran, R. S. , Heighington, C. S. , Starostina, N. G. , Anderson, J. W. , Owen, D. L. , Vasudevan, S. , & Kipreos, E. T. (2016). The ubiquitin ligase CRL2ZYG11targets cyclin B1 for degradation in a conserved pathway that facilitates mitotic slippage. Journal of Cell Biology, 215(2), 151–166. 10.1083/jcb.201601083 27810909 PMC5084644

[mgg31375-bib-0002] Barber, J. C. K. (2018). Reassignment of *HMX1* indicates copy number variation within 4p16.1 may be an alternative cause of oculoauricular phenotypes. American Journal of Medical Genetics Part A, 176(9), 2034–2036. 10.1002/ajmg.a.40385 30055074

[mgg31375-bib-0003] Barisic, I. , Odak, L. , Loane, M. , Garne, E. , Wellesley, D. , Calzolari, E. , … Tucker, D. (2014). Prevalence, prenatal diagnosis and clinical features of oculo‐auriculo‐vertebral spectrum: A registry‐based study in Europe. European Journal of Human Genetics, 22(8), 1026–1033. 10.1038/ejhg.2013.287 24398798 PMC4350601

[mgg31375-bib-0004] Beleza‐Meireles, A. , Hart, R. , Clayton‐Smith, J. , Oliveira, R. , Reis, C. F. , Venâncio, M. , … Tassabehji, M. (2015). Oculo‐auriculo‐vertebral spectrum: Clinical and molecular analysis of 51 patients. European Journal of Medical Genetics, 58(9), 455–465. 10.1016/j.ejmg.2015.07.003 26206081

[mgg31375-bib-0005] Berenguer, M. , Tingaud‐Sequeira, A. , Colovati, M. , Melaragno, M. I. , Bragagnolo, S. , Perez, A. B. A. , … Rooryck, C. (2017). A novel de novo mutation in MYT1, the unique OAVS gene identified so far. European Journal of Human Genetics, 25(9), 1083–1086. 10.1038/ejhg.2017.101 28612832 PMC5558169

[mgg31375-bib-0006] Bragagnolo, S. , Colovati, M. E. S. , Souza, M. Z. , Dantas, A. G. , F de Soares, M. F. , Melaragno, M. I. , & Perez, A. B. (2018). Clinical and cytogenomic findings in OAV spectrum. American Journal of Medical Genetics, Part A, 176(3), 638–648. 10.1002/ajmg.a.38576 29368383

[mgg31375-bib-0007] Coberly, S. , Lammer, E. , & Alashari, M. (1996). Retinoic acid embryopathy: Case report and review of literature. Pediatric Pathology & Laboratory Medicine, 16(5), 823–836. 10.1080/15513819609169308 9025880

[mgg31375-bib-0008] Derbent, M. , Y?lmaz, Z. , Baltac?, V. , Sayg?l?, A. , Varan, B. , & Tokel, K. (2003). Chromosome 22q11.2 deletion and phenotypic features in 30 patients with conotruncal heart defects. American Journal of Medical Genetics, 116A(2), 129–135. 10.1002/ajmg.a.10832 12494430

[mgg31375-bib-0009] Dos Santos, P. A. C. , de Oliveira, S. F. , Freitas, E. L. , Safatle, H. P. N. , Rosenberg, C. , Ferrari, I. , & Mazzeu, J. F. (2014). Non‐overlapping 22q11.2 microdeletions in patients with oculo‐auriculo‐vertebral spectrum. American Journal of Medical Genetics, Part A, 164(2), 551–553. 10.1002/ajmg.a.36231 24311469

[mgg31375-bib-0010] Glineur, R. , Louryan, S. , Lemaître, A. , Evrard, L. , Rooze, M. , & De Vos, L. (1999). Cranio‐facial dysmorphism: Experimental study in the mouse, clinical applications. Surgical and Radiologic Anatomy, 21(1), 41–47. 10.1007/BF01635051 10370992

[mgg31375-bib-0011] Goldenhar, M. (1952). Associations malformatives de l’oeil et de l’oreille, en particulier le syndrome dermoïde epibulbaire‐appendices auriculaires‐fistula auris congenita et ses relations avec la dysostose mandibulo‐faciale. Journal De Génétique Humaine, 243–282.

[mgg31375-bib-0012] Gorlin, R. J. , Cohen, M. , & Hennekam, R. C. M. (2001). Branchial arch and oro‐acral disorders In Syndromes of the Head and Neck (pp. 790–798). USA: Oxford University Press.

[mgg31375-bib-0013] Gorlin, R. J. , Jue, K. L. , Jacobsen, U. , & Goldschmidt, E. (1963). Oculoauriculovertebral dysplasia. The Journal of Pediatrics, 63(5), 991–999. 10.1016/S0022-3476(63)80233-4 14071056

[mgg31375-bib-0014] Lammer, E. J. , Chen, D. T. , Hoar, R. M. , Agnish, N. D. , Benke, P. J. , Braun, J. T. , … Sun, S. C. (1985). Retinoic acid embryopathy. The New England Journal of Medicine, 313(14), 837–841. 10.1056/NEJM198510033131401 3162101

[mgg31375-bib-0015] Liu, J. , Vasudevan, S. , & Kipreos, E. T. (2004). CUL‐2 and ZYG‐11 promote meiotic anaphase II and the proper placement of the anterior‐posterior axis in C. elegans. Development, 131(15), 3513–3525. 10.1242/dev.01245 15215209

[mgg31375-bib-0016] Lopez, E. , Berenguer, M. , Tingaud‐Sequeira, A. , Marlin, S. , Toutain, A. , Denoyelle, F. , … Rooryck, C. (2016). Mutations in MYT1, encoding the myelin transcription factor 1, are a rare cause of OAVS. Journal of Medical Genetics, 53(11), 752–760. 10.1136/jmedgenet-2016-103774 27358179

[mgg31375-bib-0017] Mendelsohn, B. A. , Yin, C. , Johnson, S. L. , Wilm, T. P. , Solnica‐Krezel, L. , & Gitlin, J. D. (2006). Atp7a determines a hierarchy of copper metabolism essential for notochord development. Cell Metabolism, 4(2), 155–162. 10.1016/j.cmet.2006.05.001 16890543

[mgg31375-bib-0018] Mondal, D. , Shenoy, R. S. , & Mishra, S. (2017). Retinoic acid embryopathy. International Journal of Applied and Basic Medical Research, 7(4), S72–S77. 10.4103/ijabmr.IJABMR 29308367 PMC5752814

[mgg31375-bib-0019] Poswillo, D. (1975). Causal mechanisms of craniofacial deformity. British Medical Bulletin, 31(2), 101–106. 10.1093/oxfordjournals.bmb.a071260 1164598

[mgg31375-bib-0020] Raldúa, D. , & Babin, P. J. (2007). BLT‐1, a specific inhibitor of the HDL receptor SR‐BI, induces a copper‐dependent phenotype during zebrafish development. Toxicology Letters, 175(1–3), 1–7. 10.1016/j.toxlet.2007.08.007 17890024

[mgg31375-bib-0021] Rengasamy Venugopalan, S. , Farrow, E. , Sanchez‐Lara, P. A. , Yen, S. , Lypka, M. , Jiang, S. , & Allareddy, V. (2019). A novel nonsense substitution identified in the AMIGO2 gene in an Occulo‐Auriculo‐Vertebral spectrum patient. Orthodontics and Craniofacial Research, 22(S1), 163–167. 10.1111/ocr.12259 31074142

[mgg31375-bib-0022] Sandoval, I. T. , Manos, E. J. , Van Wagoner, R. M. , Delacruz, R. G. , Edes, K. , Winge, D. R. , … Jones, D. A. (2013). Juxtaposition of chemical and mutation‐ induced developmental defects in zebrafish reveal a novel copper‐chelating activity for kalihinol F. Chemistry & Biology, 20(6), 753–763. 10.1016/j.chembiol.2013.05.008 23790486 PMC3715381

[mgg31375-bib-0023] Sonneville, R. , & Gönczy, P. (2004). zyg‐11 and cul‐2 regulate progression through meiosis II and polarity establishment in C. elegans. Development, 131(15), 3527–3543. 10.1242/dev.01244 15215208

[mgg31375-bib-0024] Stoll, C. , Viville, B. , Treisser, A. , & Gasser, B. (1998).Oculoauriculovertebral Spectrum, 349(February), 345–349. 10.1002/(sici)1096-8628(19980724)78:4<345::aid-ajmg8>3.0.co;2-k 9714437

[mgg31375-bib-0025] Vendramini‐Pittoli, S. , & Kokitsu‐Nakata, N. M. (2009). Oculoauriculovertebral spectrum: Report of nine familial cases with evidence of autosomal dominant inheritance and review of the literature. Clinical Dysmorphology, 18(2), 67–77. 10.1097/MCD.0b013e328323a7dd 19305190

[mgg31375-bib-0026] Wang, R. , Martínez‐Frías, M. L. , & Graham, J. M. (2002). Infants of diabetic mothers are at increased risk for the oculo‐auriculo‐vertebral sequence: A case‐based and case‐control approach. Journal of Pediatrics, 141(5), 611–617. 10.1067/mpd.2002.128891 12410187

[mgg31375-bib-0027] Xu, J. , Fan, Y. S. , & Siu, V. M. (2008). A child with features of Goldenhar syndrome and a novel 1.12 Mb deletion in 22q11.2 by cytogenetics and oligonucleotide array CGH: Is this a candidate region for the syndrome? American Journal of Medical Genetics, Part A, 146(14), 1886–1889. 10.1002/ajmg.a.32359 18553512

[mgg31375-bib-0028] Yamaki, Y. , Kagawa, H. , Hatta, T. , Natsume, T. , & Kawahara, H. (2016). The C‐terminal cytoplasmic tail of hedgehog receptor Patched1 is a platform for E3 ubiquitin ligase complexes. Molecular and Cellular Biochemistry, 414(1–2), 1–12. 10.1007/s11010-015-2643-4 26885983

