# Supplementary material for: Functional and genetic analyses of ZYG11B provide evidences for its involvement in OAVS
Source: Mol Genet Genomic Med. 2020 Aug 1;8(10):e1375. doi: 10.1002/mgg3.1375 (PMC7549578; doi:10.1002/mgg3.1375)
Supplement: Supplementary file 1 — Table S1‐Fig S1 [file MGG3-8-e1375-s001.docx]

**Functional and genetic analyses of *ZYG11B* provide evidences for its involvement in OAVS.**

Angèle Tingaud-Sequeira^1^, Aurélien Trimouille^1,2^, Sandrine Marlin^3,4^, Estelle Lopez^1^, Marie Berenguer1, Souad Gherbi^3^, Benoit Arveiler^1,2^ , Didier Lacombe^1,2^, Caroline Rooryck^1,2^*.

**Supplemental table S1. Primers used in the study.**

| Experiment |  | Sequence (5’->3’) | Amplicon length |
| --- | --- | --- | --- |
| *ZYG11B* cloning | *Forward* | CAGGACGGAGGCTGCATG | 2336 bp |
|  | *Reverse* | CCAAAACATCAGGGAGGGTAAGA |  |
| *ZYG11B* subcloning | *Forward* | CCATCGATTCGAATTCAGGACGGAGGCTGCATG | 2366 bp |
|  | *Reverse* | GAGAGGCCTTGAATTCCAAAACATCAGGGAGGG |  |
| ZYG11B-E537* Mutagenesis | *Forward* | CCAACCACTTGTAGACACTTTATTTAAAACCAAGGGTTAGAACTC |  |
|  | *Reverse* | GAGTTCTAACCCTTGGTTTTAAATAAAGTGTCTACAAGTGGTTGG |  |
| *ZYG11B* expression | *Forward* | GAGGTGGCTGATCGACTGCT |  |
|  | *Reverse* | GCACCAGGCACTGGAGATTC | 271 bp |
| *SOX6* expression | *Forward* | CACCCCATAATGCACAACAA | 205 bp |
|  | *Reverse* | TTATCTCACGGTCCCGACTC |  |
| *zyg11* expression pattern | *Forward* | GGCTCGTGCACTTCTCAGTA | 405 bp |
|  | *Reverse* | TCCCACCGTACTGTCATTCA |  |
| *zyg11l* expression pattern | *Forward* | AGTTTTGCACAGGCTGGTGT | 445 bp |
|  | *Reverse* | TGAAGCTTGTGGATGCAGAG |  |
| *eef1a1* expression pattern | *Forward* | GATGCACCACGAGTCTCTGA | 155 bp |
|  | *Reverse* | TGATGACCTGAGCGTTGAAG |  |


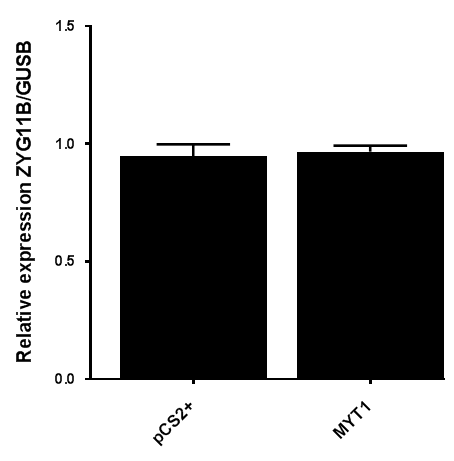


**Supplemental Figure S1: *ZYG11B* gene expression is not deregulated by *MYT1* overexpression in HeLa cells.** RT-qPCR analysis of ZYG11B expression following overexpression of either empty pCS2+ vector or pCS2+-MYT1 (Mann-Whitney test, p<0.05) (n=3).
